# Malaria in Mauritania: retrospective and prospective overview

**DOI:** 10.1186/s12936-015-0607-5

**Published:** 2015-03-04

**Authors:** Khadijetou Mint Lekweiry, Mohamed Salem Ould Ahmedou Salem, Leonardo K Basco, Sébastien Briolant, Jamaleddine Hafid, Ali Ould Mohamed Salem Boukhary

**Affiliations:** UR Génome et Milieux, Faculté des Sciences et Techniques, Université des Sciences, de Technologie et de Médecine, Nouveau Campus Universitaire, BP 5026 Nouakchott, Mauritania; Unité de Parasitologie, Institut de Recherche Biomédicale des Armées, ancienne base aérienne 217, B. P. 73, 91223 Brétigny-sur-Orge, France; Unité de Recherche sur les Maladies Infectieuses et Tropicales Emergentes, Unité de Recherche 198-Institut de Recherche pour le Développement, Faculté de Médecine La Timone, Aix-Marseille Université, 27 boulevard Jean Moulin, 13385 Marseille, France; Institut de Recherche Biomédicale des Armées, Direction interarmées du service de Santé, Laboratoire de Parasitologie, CNR du Paludisme région Antilles-Guyane, Institut Pasteur de la Guyane, Cayenne, Cedex France; Laboratoire Aliments, Environnement et Santé (LAES), Faculté des Sciences et Techniques, Université Cadi Ayyad, Marrakech, Morocco

**Keywords:** Malaria, Mauritania, Morbidity, *Plasmodium*, *Anopheles*

## Abstract

Malaria has become a major public health problem in Mauritania since the 1990s, with an average of 181,000 cases per year and 2,233,066 persons at risk during 1995–2012. This paper provides the first publicly available overview of malaria incidence and distribution in Mauritania. Information on the burden and malaria species distribution is critical for guiding national efforts in malaria control. As the incidence of malaria changes over time, regular updates of epidemiological data are necessary.

## Background

**Figure 1:**
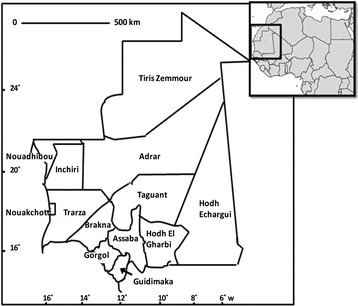
**Map of Mauritania with its 13 provinces.**

The Islamic Republic of Mauritania is situated in northwest Africa between 15 and 27° N latitude and 5 and 17° W longitude. It occupies a total surface area of 1,030,700 sq km (Figure 1). The population of Mauritania is currently estimated to be 3,378,250 inhabitants [1] (Table 1). Population pyramid shows that nearly 43.7% are under 15 years old [2]. Infant mortality rate is 77.0 per 1,000 live births [2]. The Mauritanian population belongs to several ethnic groups: Moors (including ‘white Moors’ and ‘black Moors’) and black Africans (*Poular, Wolof*, and *Soninke*). However, there are no data on the demographic weight of each ethnic group. Sedentarization and rural exodus, partly related to the periods of drought in the 1970s and 1980s, are the most significant demographic phenomena that have occurred in Mauritania since the country’s independence in 1960. Whereas the proportion of urban population was 9% in 1965, it increased to 22.7, 46.7 and 60% in 1977, 2005 and 2010, respectively. Over the same period, the nomad population rapidly decreased from 65% in 1965 to 12 and 6% in 1988 and 2000, respectively [3]. Since its independence in 1960, Mauritania has experienced intense migratory movements. Outward flows started in the 1970s mainly due to severe and frequent droughts. These early flows were mainly directed towards sub-Saharan African countries, such as Senegal, Mali, Ivory Coast, and Gambia. Inward flows, coming mainly from neighbouring countries (Senegal, Mali, Guinea Conakry, and Ivory Coast), consisted of labour migration which filled the needs of the labour market. The total number of regular immigrants in 2010 was estimated to be 99,229, corresponding to 3% of the total resident population [4]. A large inward flow of refugees and asylum seekers due to civil conflicts in African countries (i.e. Liberia, Sierra Leone, Ivory Coast, and Mali) occurred in the 1990s. Currently, Mauritania hosts 93,590 refugees, including 66,400 Malian refugees, who left their country due to the most recent conflict in Mali [5]. Within the last decade, Mauritania has also evolved into an important transit country, attracting irregular migrants from sub-Saharan African countries attempting to enter into Europe through the Canary Islands, Ceuta, and Melilla.

**Table 1:** **Population demographic data of Mauritania**

**Indicator**	**Value**	**Reference**
Population density (inhabitant/sq km)	3.4	[1]
Urban population (%)	60	[2]
Gender		
Male (%)	50.2	[2]
Female (%)	49.6	[2]
Age groups (%)		
<15 years	43.7	[2]
15-65 years	51.0	[2]
>65 years	5.3	[2]
Birth rate (%)	4.6	[2]
Mortality rate (%)	1.3	[2]
Infant mortality (%)	7.7	[2]
Growth rate	2.4	[2]
Life expectancy at birth (years)	57.5	[2]

**Figure 2:**
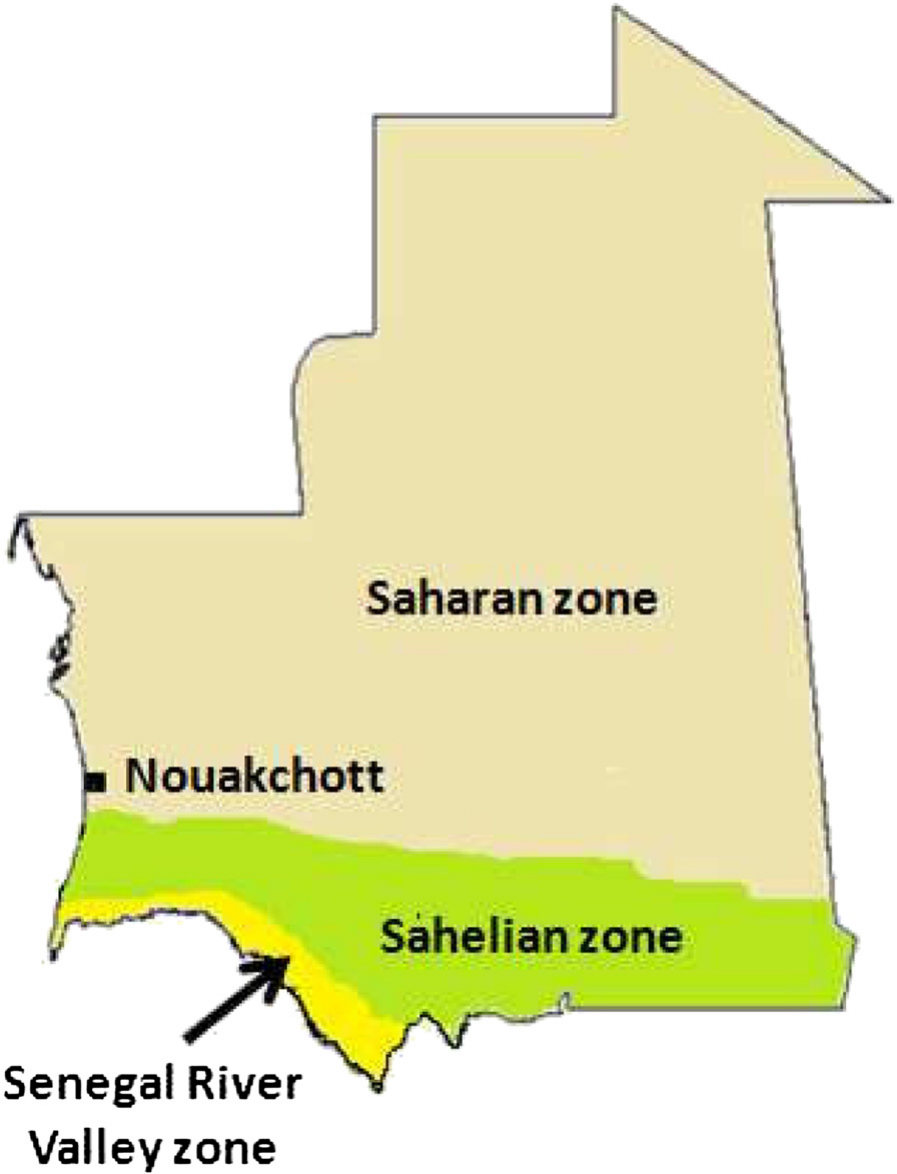
**Major agro-climatic zone of Mauritania.**

Two-thirds of the surface area of Mauritania is covered by the Sahara Desert and one-third is sub-Saharan semi-desert. It has a low annual rainfall that increases from north to south, one rainy season from June/July to September/October, depending on the year and/or region, and a dry season (‘cold dry season’ between November to March and ‘hot dry season’ from March to June). Accordingly, the country is geographically divided into three ecological areas (Figure 2): arid Saharan zone in the north, Sahelian zone in the intermediate region and the Senegal River valley zone in the extreme south. The arid zone covers the land surface below 150 mm isohyets. It corresponds to the Saharan climate characterized by the presence of several oases that have been established for centuries, mainly in Adrar and Tagant regions. The Sahelian zone, made up of steppe and savannah grassland, is characterized by annual rainfall of 100–300 mm. In this region, the inhabitants practice animal breeding and rain-fed agriculture. The Senegal River Valley region, sometimes known as Shemama, is a narrow belt of land that extends north and east of the Senegal River. Rainfall is higher than in other regions, ranging from 300–500 mm annually. This rainfall, combined with annual flooding of the river, provides the basis for agriculture with a potential farmland estimated at 135,000 hectares. The main and most important permanent water source in Mauritania is the Senegal River that runs along the southwestern common border with Senegal.

### Malaria distribution and causative agents

**Figure 3:**
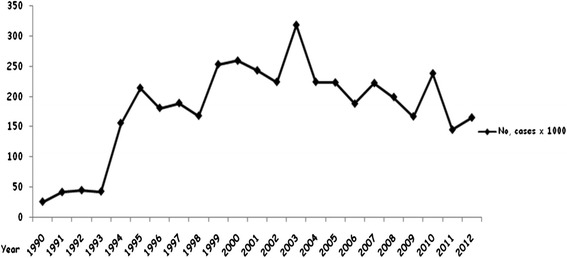
**Number of reported malaria cases from 1990 to 2012 in Mauritania.**

The history of documented malaria cases in Mauritania is relatively recent. The official statistics, mostly based on clinical diagnosis without laboratory confirmation, show an increase of malaria cases during the last two decades from 26,933 in 1990 to 165,834 in 2012 [6] with an average of 181,000 cases per year (Figure 3). The disease is currently the leading cause of morbidity and mortality and the first cause of consultation and hospitalization in eight of 13 regions of the country. Indeed, during the transmission season (August-November), 60% of hospital admissions are due to presumptive diagnosis of malaria, which represents, on average, 25% of morbidity and over 39% of mortality at health facilities [7]. Moreover, malaria is considered the third motive of outpatient consultations at national level, after acute respiratory infections and diarrhoea [7].

**Figure 4:**
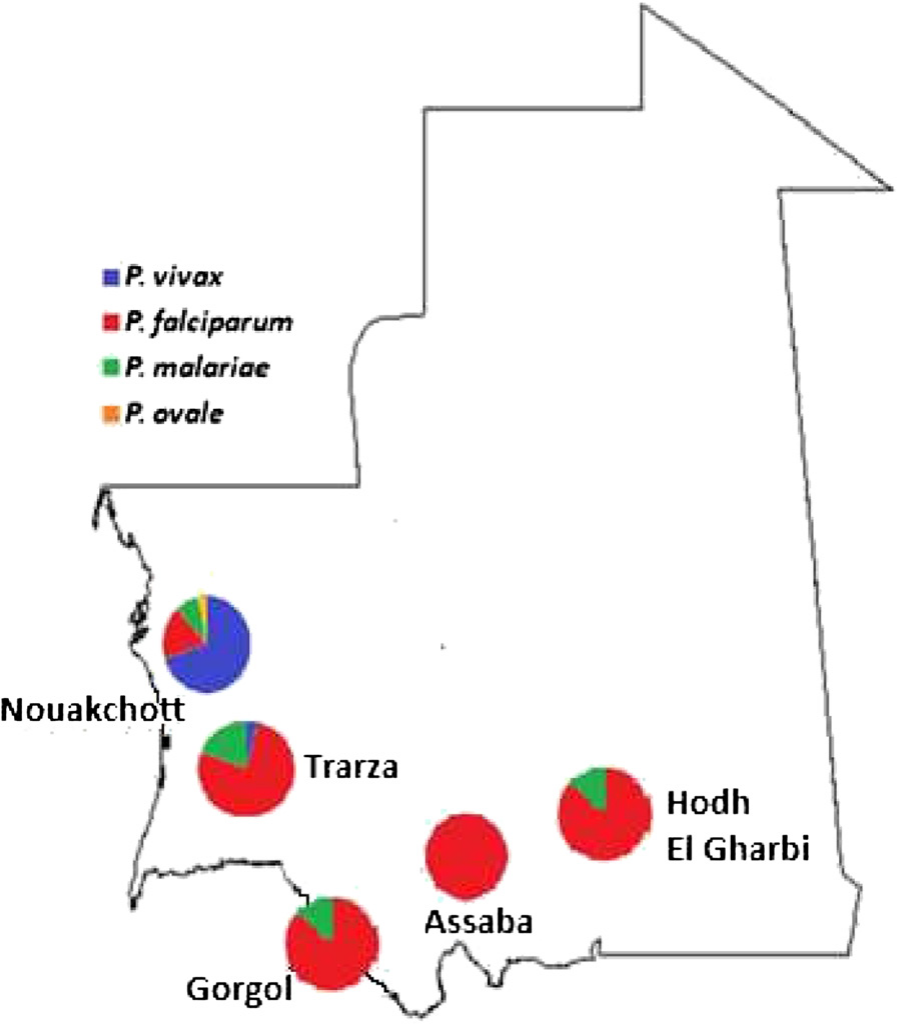
**Distribution of** ***Plasmodium*** **spp. based on microscopy in Mauritania.**

Studies conducted in different regions reported the presence of four malaria species in Mauritania (Figure 4), namely *Plasmodium falciparum*, *Plasmodium vivax*, *Plasmodium ovale*, and *Plasmodium malariae* [8-12]. *Plasmodium falciparum* was the dominant species, diagnosed in 90% of reported clinical malaria cases. The recorded infection rates were variable depending on the season, region and rainfall. In the first published report from the southern region of Gorgol, Sautet *et al.* [8] reported 180 positive malaria cases among 307 febrile patients, corresponding to a malaria prevalence rate of 59%. *Plasmodium falciparum* was detected in 133 (43.3%) of 307 examined slides, followed by *P. malariae* with (10.7%) and *P. vivax* (4.5%). In another study conducted in southern Mauritania, Escudie and Hamon [13] reported two human malaria species with a predominance of *P. falciparum* over *P. malariae*. Three years later, a survey conducted in the cities of Atar in northern Mauritania and Kaedi, Kiffa, Kankoussa, Aioun, and Tamchakett in southern Mauritania, noted the presence of only *P. falciparum* and *P. malariae*, with an infection rate of 7% [10]. The sero-prevalence of malaria was also studied in schoolchildren throughout the country. It revealed a sero-prevalence of 8%, indicating a recent history of infection with *P. falciparum* in Ksar Torchane (Saharan part of the country), 58% in Boutilimit (Sahelian region), and 34-45% in the Sahelian region of Assaba region. The survey also reported a malaria serological prevalence of 12% in March 1973 (during the dry season) and 14% in November 1972 (just after the rainy season) among schoolchildren in Nouadhibou, a coastal city in northwestern Mauritania [14]. In a survey conducted in 1984, one year following the construction of Foum Legleita Dam in the Gorgol region, Baudon *et al.* [15] reported a very low level of anti-malarial antibodies using indirect immunofluorescence. Of the surveyed population, 73% had anti-malarial antibody titres of less than 1/40 and only 4% of them displayed antibodies titres of 1/640 or above.

More recently, studies conducted in Nouakchott, the capital city of Mauritania, showed that infection due to malaria has increased with a high rate of infection due to *P. vivax*. In 2003, Cortes *et al.* [11] found 77 positive slides out of 446 blood smears collected from febrile patients attending health facilities in Nouakchott. Malaria infection rate was 18.5% (77/446), with 61.8% due to *P. falciparum* and 35.5% due to *P. vivax*. The authors also found nine malaria cases (seven due to *P. falciparum* and two due to *P. vivax*), which were considered as autochthonous. Moreover, Mint Lekweiry *et al.* [12] reported a malaria prevalence of 25.7% (61/237) among febrile patients attending three health facilities in Nouakchott in 2007. The majority of these cases were due to *P. vivax* (43/61, 70.5%), followed by *P. ovale* (15/61, 24.6%) and *P. falciparum* (3/61, 4.9%) by microscopy. PCR performed on these samples confirmed all *P. vivax* and *P. falciparum* infections, but cases initially diagnosed to be *P. ovale* infections by microscopy turned out to be *P. vivax* by PCR. Therefore, after PCR correction, the proportion of *P. vivax* to *P. falciparum* was 58/61 (95.1%), and no *P. ovale* or *P. malariae* malaria parasites were detected by PCR. This study also revealed that among 237 recruited febrile patients, 231 were clinically diagnosed and treated as malaria cases and that false positive constituted 73.6% of the clinically diagnosed malaria. This finding highlights the importance of the timely use of microscopy and, in case of unavailability of microscopes and/or well-trained microscopists, an alternative reliable laboratory tool, in particular rapid diagnostic tests for malaria, will play a major role in establishing the correct diagnosis and appropriate treatment.

In the follow-up study performed in 2009–2010, the same authors found a malaria prevalence of 34.9% (105 of 301 febrile patients) among children born and residing permanently in Nouakchott [16]. Among these patients with malaria confirmed by microscopy, rapid diagnostic test and PCR, 102 of 105 (97.1%) infections were due to *P. vivax* and 2.9% (three of 105) were caused by *P. falciparum*. The results of this 2009–2010 study confirmed malaria prevalence among febrile patients and distribution of *Plasmodium* spp. in Nouakchott. Moreover, 54 of the *P. vivax*-infected children had never travelled outside Nouakchott, suggesting that malaria transmission occurs within the city and that *P. vivax* is the principal causative agent.

In general, while malaria diagnosis, based on thick and thin smears, previously reported the presence of four malaria species in the country [8-11], more recent data obtained using nested PCR showed the presence of only *P. vivax* and *P. falciparum* in Nouakchott and southern Mauritania [12,16,17]. Furthermore, malaria affects all age groups in the country [7,11]. However, data on morbidity and mortality associated with this disease are underestimated due to limited health information and insufficient resources to confirm diagnosis with microscopy (or rapid diagnostic test for malaria) in most health centres. Most febrile patients tend to practice self-medication for fever at home or prefer consulting private health sectors, for which there are no health data in the country. It is worth noting that despite the publication of several papers demonstrating *P. vivax* endemicity in Nouakchott, the World Malaria Report 2013 erroneously indicates that Mauritania is free of *P. vivax,* demonstrating a gap in the quality of national data submitted to WHO [6].

### Population structure and drug resistance of malaria parasites

In 1991, during the survey of anti-malarial drug resistance in West Africa, Guiguemde *et al.* [18] reported that only Mauritania is still spared from chloroquine resistance. Three years later, chloroquine-resistant *P. falciparum* was detected in the autochthonous Mauritanian population. Gasquet *et al.* [19] investigated clinical response to chloroquine among 37 febrile patients infected with *P. falciparum* attending the health facility in Kiffa (Assaba region) with the following characteristics: a minimum parasite density of 1,000 asexual parasites/μl of blood, absence of chloroquine in the urine, and no recent history of treatment with other anti-malarial agents. The patients received a standard dose of 25 mg base of chloroquine per kg body weight over three days and were followed until day 7. Six patients were lost to follow-up. Three (9.7%) of the 31 enrolled patients had recurrent asexual parasitaemia, and one patient remained febrile on day 7, suggesting clinical resistance to chloroquine.

Jelinek *et al.* [20] combined a clinical study on chloroquine efficacy, conducted in 1998, with an analysis of molecular markers for drug resistance, *P. falciparum* chloroquine resistance transporter (*pfcrt*) and *P. falciparum* multidrug-resistance gene 1 (*pfmdr1*), after an unusually heavy rainfall in Aioun and Kobeni, southern Mauritania. Clinical chloroquine resistance was detected in 33 of 85 (38.8%; 19 (22.3%) RI resistance and 14 (16.5%) RII/RIII resistance) *P. falciparum*-infected patients followed until day 14. Molecular analysis showed a sensitivity for detection of clinical resistance of 60.6% and a specificity of 65.3% for *pfmdr1* 76-tyrosine mutant allele while Lys76Thr mutation in *pfcrt* gene showed a very high sensitivity (100%) but low specificity (65.4%) to detect clinical resistance. The combination of mutations in both genes decreased the sensitivity to 60.6% while specificity increased to 76.9%. The authors concluded that *pfcrt*, but not *pfmdr1*, may be useful to monitor chloroquine resistance in Mauritania.

Molecular analysis of resistance to antifolates (pyrimethamine and sulphadoxine) was performed by Eberl *et al.* [21] using the same blood samples collected in Aioun and Kobeni in an earlier study [20]. These authors examined 162 *P. falciparum* isolates collected in Hodh Elgharbi region (southeast Mauritania) from patients recruited at two medical centres in Aioun, the regional capital city and Kobeni, a small city near the border with Mali, for point mutations in dihydrofolate reductase (*dhfr*) (codons 16, 51, 59, 108, and 164) and dihydropteroate synthase (*dhps*) genes (codons 436, 437, 540, 581 and 613). *Dhfr* mutations were found at a low prevalence both in Aioun and Kobeni (18.6% of samples from Aioun and 12.6% of samples from Kobeni showed at least one mutation), whereas a high prevalence of *dhps* mutations was noted among isolates from Aioun (74.6%) and Kobeni (63.4%), respectively. These results suggested that sulphadoxine-pyrimethamine may be effective for intermittent preventive treatment in pregnant Mauritanian women (the only current indication of sulphadoxine-pyrimethamine in Mauritania), but more clinical data are required to confirm the molecular findings.

In 2012, Mint Lekweiry *et al.* [22] reported a low prevalence of mutants in the *P. vivax* population isolated from 110 malaria-infected patients in Nouakchott. Indeed, the majority of the isolates (n = 100, 90.9%) were genotyped as wild-type *P. vivax dhfr* (*pvdhfr*), while the remaining ten isolates carried the Ser58Arg and Ser117Asn double mutations (homologues of Cys59Arg and Ser108Asn in *P. falciparum dhfr*). All isolates had the wild-type *P. vivax dhps* (*pvdhps*) alleles. For *P. vivax* multidrug resistance gene 1 (*pvmdr1*), 75 of 103 (73%) had the wild-type Tyr976, and 28 (27%) carried the mutant Phe976. Most (98%) carried the mutant Leu1076 codon. Of 105 isolates, 102 (97%) had one gene copy and three (3%) had two copies of the *pvmdr1* gene. *Pvmdr1* mutations have been suggested to be associated with chloroquine resistance in *P. vivax*, whereas an increased number of copy number has been associated with resistance to mefloquine [23,24].

Studies on the genetic structure of *Plasmodium* spp. populations in Mauritania are very limited. Jordan *et al.* [17] genotyped 173 *P. falciparum* isolates, collected in Aioun and Kobeni in 1998 as part of the clinical study on chloroquine efficacy, for three polymorphic genetic markers: merozoite surface protein 1 (*msp1*) and merozoite surface protein 2 (*msp2*), and glutamate-rich protein (*glurp*). The differences in the population structure between the two study areas were seen in both the mean number of distinct parasite populations per infection (1.57 for Aioun and 2.34 for Kobeni) and their distribution in different allelic groups. For *msp2*, the differences in the number of size variations and amplified fragments were observed in the *msp2* FC27 allelic family. Isolates from Aioun showed one or two bands with six different sizes, while amplification of the samples from Kobeni produced up to four bands with 18 different band sizes. However, analysis of *glurp* provided no further conclusive information because all samples showed a single band of the same size, suggesting limited heterogeneity of *P. falciparum* populations circulating in this region. More recently, Ould Ahmedou Salem *et al.* [25] examined and compared the genetic diversity of *msp1* block 2 region among *P. falciparum* isolates in Nouakchott, the capital city of Mauritania, and Hodh Elgharbi region (southeast of Mauritania). K1, Mad20 and Ro33 msp1 alleles were identified with 27 different sized alleles. Infection with multiple *P. falciparum* populations was observed in the two regions (93/113; 82.3%), as well as a high multiplicity of infection (3.2 genotypes per infection), reflecting both the high endemicity level and malaria transmission in the study areas.

### Malaria vectors

The first published studies on malaria vectors date back to the 1940s [8], followed by those of Maffi [26] and Hamon *et al.* [27]. These authors reported the presence of the following 12 *Anopheles* species and subspecies in Mauritania: *Anopheles funestus*, *Anopheles gambiae s.l*., *Anopheles pharoensis*, *Anopheles rufipes*, *Anopheles melas*, *Anopheles dhtali*, *Anopheles rhodesiensis*, *Anopheles coustani*, *Anopheles ziemmani*, *Anopheles pretoriensis*, *Anopheles squamosus*, and *Anopheles demilloni*. Among these anopheline species, only *An. gambiae* and *An. funestus* are known to be major malaria vectors in Africa. In Mauritania, *An. gambiae s.l*. appears to be the dominant malaria vector [8,28]. However, until today, their distribution in the country has not been established (Table 2).

**Table 2:** **Distribution of the** ***Anopheles gambiae*** **complex and** ***Anopheles funestus*** **group in Mauritania**

**Region**	***Anopheles sp.***	**Reference**
Adrar	No data	
Assaba	*An. arabiensis; An. gambiae; An. gambiae s.l.* and *An. funestus*	[26-28]
Brakna	*An. gambiae* ^*1*^ *; An. gambiae s.l.* and *An. funestus*	[8,29]
Gorgol	*An. gambiae s.l.* and *An. funestus*	[8]
Guidimaka	*An. gambiae s.l.*	[27]
Hodh Echargui	*An. gambiae s.l.*	[27]
Hodh Elgharbi	*An. gambiae*	[28]
Inchiri	No data	
Nouadhibou	No data	
Nouakchott	*An. arabiensis; An. gambiae s.l.*	[29,12,30]
Tagant	*An. gambiae s.l.*	[28]
Trarza	*An. gambiae s.l.* and *An. funestus*	[28]
Tiris zemmour	No data	

Recently, Dia *et al.* [28] studied the distribution, host preference and infection rates of three malaria vectors, *Anopheles arabiensis*, *An. pharoensis* and *An. funestus*, in 21 localities in five regions in Mauritania, namely Trarza, Brakna, Assaba, Hodh Elgharbi and Tagant. They collected 647 anopheline specimens, among which *An. gambiae s.l*. was the most commonly identified species (92%), followed by *An. pharoensis* (5%) and *An. funestus* (3%). *Anopheles gambiae* was collected in all localities except in Moudjeria (north of the country) where there was no mosquito captured during the study period. The molecular identification of the *An. gambiae* complex revealed the predominance of *An. arabiensis* and the presence of *An. gambiae* (M form) in Assaba, Brakna and Hodh Elgarbi regions. In Nouakchott, the presence of anopheline mosquitoes (*An. gambiae s.l.*) was first reported in Toujounin district [29]. More recently, Mint Lekweiry *et al.* [12] demonstrated the presence of *An. arabiensis* and *An. pharoensis* in Dar Naim district in Nouakchott. Ould Ahmedou Salem *et al.* [30] have recently shown, for the first time, the existence of *An. gambiae s.l*. larval habitats in Nouakchott and described their ecological and physico-chemical characteristics. They reported that water bodies consisting of water discharged from standpipes and household drinking water tanks served as *Anopheles* spp. larval habitats. Using multivariate regression analyses, it was further shown that salinity up to 0.1 g/l and shaded habitats were protective factors against high larvae density in breeding sites and that pH up to 7.61 was a risk factor to develop high larvae density in these breeding sites. However, studies on bionomics, epidemiology of anopheline species, and their involvement in malaria transmission remain poorly understood although the presence of *P. falciparum* and *P. vivax* sporozoites was demonstrated in the salivary glands of *An. arabiensis* collected in Mauritania [28,31].

### National malaria control programme: policy strategy and plans

The Mauritanian Ministry of Health executes malaria control through the National Malaria Control Programme, which has elaborated a quadrennial strategic plan against malaria epidemics and endemicity. The stated goal of the latest plan for 2011–2015 is to reduce, by 2015, malaria-related morbidity and mortality from 17.5% and 0.39 per 100,000 inhabitants in 2011 to 10.9% and 0.15 per 100,000 inhabitants in 2015, respectively [2]. The strategies for achieving this goal are based on the establishment of a monitoring system, early detection of malaria epidemics, reliable laboratory-based diagnosis and appropriate and effective treatment of malaria cases, a system of prevention of and appropriate response to malaria epidemics, and collection of factual evidence on malaria epidemics. The promotion and distribution of insecticide-treated bed nets (ITNs) among populations living in endemic areas, particularly during the transmission season, the use of sulphadoxine-pyrimethamine for intermittent preventive treatment (IPT) to control malaria during pregnancy, particularly during the first and second pregnancies, are the main objectives of this strategy.

As of 2014, ITNs have been distributed free of charge to the major risk groups in southern Mauritania, i.e. among pregnant women, and IPT has been effectively implemented since 2008 [32]. However, intradomiciliary residual spraying and larval control have not been recommended by the Mauritanian National Malaria Control Programme. Laboratory diagnosis of malaria is free of charge for febrile patients of all ages in the public sector and is mainly based on the use of rapid diagnostic test, and not microscopy, due to the shortage of microscopes under working condition and well-trained microscopists. In addition, it is recommended for travellers visiting endemic areas in the south to use chemoprophylaxis (atovaquone-proguanil, doxycycline or mefloquine) starting from ten to 21 days before departure, during their stay and three weeks following their return from these regions [33]. Furthermore, a national malaria day has been organized annually since 1996 by the Ministry of Health to inform and educate the public about the importance and risks of malaria.

It is worth noting that in 2006, following the emergence of chloroquine-resistant *P. falciparum* in West Africa, particularly in the Senegal River Basin, the Mauritanian Government decided to replace chloroquine with artemisinin-based combination therapy (ACT). This policy change was principally based on clinical studies conducted in Senegal and extrapolated to the Mauritanian situation. Regardless of the parasite species involved, the treatment of uncomplicated malaria is currently based on artesunate-amodiaquine as the first-line treatment, artemether-lumefantrine as the second choice, and quinine as the third-line drug [7]. Monotherapies using artemisinin derivatives have been withdrawn, and ACT is provided free of charge to laboratory-confirmed malaria-infected patients of all ages in the public sector. For the treatment of *P. vivax* malaria, the National Malaria Control Programme recommends the use of primaquine for radical treatment if glucose-6-phosphate dehydrogenase (G6PD) test is normal. This drug policy has not yet been implemented in the field. As part of future prospective studies aiming to develop an evidence-based national anti-malarial drug policy, the National Malaria Control Programme and the University of Sciences, Technology and Medicine in Nouakchott have been collaborating since 2012 to conduct clinical studies in several sentinel sites in the country to validate and, if needed, modify the current anti-malarial treatment guidelines.

## Conclusion

Mauritania faces several challenges in the management of malaria, including limited financial resources, crisis in human resources, shortage of health workers and health structures with reliable diagnostic facilities, insufficient epidemiological data on parasite distribution and malaria vectors, and regular epidemics. Entomological studies are currently being conducted at several sentinel sites in different epidemiological strata in the country to capture and collect mosquito larvae and adults, identify predominant *Anopheles* spp. in each study site, characterize physico-chemical factors in the breeding sites, determine whether adult female *Anopheles* mosquitoes are infective, and assess their sensitivity to insecticides. In parallel, malaria burden and drug resistance are being assessed in these sentinel sites to determine the prevalence of laboratory-confirmed malaria among febrile patients and their response to ACT. Research capacity building and training of laboratory technicians and general practitioners are integral parts of these on-going field activities. The introduction of ACT is another challenge facing Mauritania. One of the key issues is the cost of ACT, which is 20 times higher than the cost of conventional therapy, such as quinine ($2.44 per adult dose). Population migration from sub-Saharan endemic countries constitutes another challenge where no official measures for controlling malaria prevalence among these migrants exist. Mauritania still does not have a reliable database on malaria epidemiology. A nationwide malaria indicator survey is being planned to fill this gap in 2015. Updated information and maps on malaria burden and *Plasmodium* species distribution are critical for guiding national efforts to improve laboratory diagnosis, implement rapid and effective treatment, and coordinate and plan malaria elimination from the country in the near future. As malaria incidence changes over time, regular updates of epidemiological data are necessary.

## Abbreviations

PCR: Polymerase chain reaction
*PFCRT*: *Plasmodium falciparum* chloroquine resistance transporter
*PFMDR1*: *Plasmodium falciparum* multidrug-resistance gene 1
Lys: Lysine
Thr: Threonine
*DHFR*: Dihydrofolate reductase
*DHPS*: Dihydropteroate synthase
Asn: Asparagine
Arg: Arginine
Leu: Leucine
*MSP1*: Merozoite surface protein 1
*MSP2*: Merozoite surface protein 2
*GLURP*: Glutamate-rich protein
*PVDHFR*: *Plasmodium vivax* dihydrofolate reductase
Ser: Serine
Cys: Cysteine
*PVDHP*: *Plasmodium vivax* dihydropteroate synthase
*PVMDR1*: *Plasmodium vivax* multidrug resistance gene 1
Tyr: Tyrosine
Phe: Phenylalanine
ITN: Insecticide-treated bed nets
IPT: Intermittent preventive treatment
ACT: Artemisinin-based combination therapy

## References

[1] Office National de Statistique (2013). Annuaire statistique 2012.

[2] Ministère de la Santé. Plan National de Développement Sanitaire. (2012–2020), Mauritanie 2011, p. 135.

[3] Cheikh Malainine ML (2001). Recensement général de la population et de l’habitat 2000 en Mauritanie: particularité du milieu nomade.

[4] United Nations. Trends in International Migrant Stock: The 2008 Revision (United Nations database, POP/DB/MIG/Stock/Rev.2008). (http://esa.un.org/migration/index.asp?panel=2). 2009.

[5] UNHCR. UNHCR Global Report 2013, Mauritania. (http://www.unhcr.org/539809f6b.html).

[6] WHO (2013). World malaria report 2013.

[7] Ministère de la Santé. Programme national de lutte contre le paludisme: politique et stratégies nationales de lutte contre le paludisme (2011–2015). Mauritanie 2011.

[8] Sautet J, Ranque J, Vuillet F, Vuillet J (1948). Quelques notes parasitologiques sur le paludisme et l’anophélisme en Mauritanie. Med Trop (Mars).

[9] Hudleston J. Programme de prééradication du paludisme, Kaédi, Mauritanie 1961. WHO/AFR/MAL/74, 1961, p. 17.

[10] Barbié Y, Timbila R (1964). Notes sur le paludisme en République Islamique de Mauritanie. Med Trop (Mars).

[11] Cortes H, Morillas-Marquez F, Valero A (2003). Malaria in Mauritania: the first cases of malaria endemic to Nouakchott. Trop Med Int Health.

[12] Mint Lekweiry K, Ould Abdallahi M, Ba H, Arnathau C, Durand P, Trape JF (2009). A preliminary study of malaria incidence in Nouakchott. Mauritania Malar J.

[13] Escudie A, Hamon J (1961). Le paludisme en Afrique Occidentale d’expression française. Med Trop (Mars).

[14] Monjour L, Richard-Lenoble D, Palminteri R, Daniel Ribeiro C, Alfred C, Gentilini M (1984). A sero-epidemiological survey of malaria in desert and semi-desert regions of Mauritania. Ann Trop Med Parasitol.

[15] Baudon D, Robert V, Darriet F, Huerre M (1986). Impact de la construction d’un barrage avec retenue d’eau sur la transmission du paludisme (enquête paludologique menée dans le Sud-Est de la Mauritanie). Bull Soc Path Exot.

[16] Mint Lekweiry K, Basco LK, O Ahmedou Salem MS, Hafid JE, Marin-Jauffre A, O Weddih A (2011). Malaria pevalence and morbidity among children reporting at health facilities in Nouakchott, Mauritania. Trans R Soc Trop Med Hyg.

[17] Jordan S, Jelinek T, Aida AO, Peyerl-Hoffmann G, Heuschkel C, El valy AO (2001). Population structure of *Plasmodium falciparum* isolates during an epidemic in southern Mauritania. Trop Med Int Health.

[18] Guiguemde TR, Gbary AR, Ouedraogo JB, Gayibor A, Lamizana L, Maiga AS (1991). Point actuel sur la chimiorésistance du paludisme des sujets autochtones dans les Etats de l’OCCGE (Afrique de l’Ouest). Ann Soc Belg Med Trop.

[19] Gasquet M, Delmont J, Le Bras J, Delmas F, Capdegelle P, Timon-David P (1995). Chloroquine-resistant falciparum malaria in Mauritania. Lancet.

[20] Jelinek T, Aida AO, Peyerl-Hoffmann G, Jordan S, Mayor A, Heuschkel C (2002). Diagnostic value of molecular markers in chloroquine resistant falciparum malaria in southern Mauritania. Am J Trop Med Hyg.

[21] Eberl KJ, Jelinek T, Aida AO, Peyerl-Hoffmann G, Heuschkel C, El Valy AO (2001). Prevalence of polymorphisms in the dihydrofolate reductase and dihydropteroate synthetase genes of *Plasmodium falciparum* isolates from southern Mauritania. Trop Med Int Health.

[22] Mint Lekweiry K, Ould Mohamed Salem Boukhary A, Gaillard T, Wurtz N, Bogreau H, Hafid JE (2012). Molecular surveillance of drug-resistant *Plasmodium vivax* using *pvdhfr*, *pvdhps* and *pvmdr1* markers in Nouakchott, Mauritania. J Antimicrob Chemoth.

[23] Brega S, Meslin B, de Monbrison F, Severini C, Gradoni L, Udomsangpetch R (2005). Identification of the *Plasmodium vivax* mdr-like gene (*pvmdr1*) and analysis of single-nucleotide polymorphisms among isolates from different areas of endemicity. J Infect Dis.

[24] Suwanarusk R, Chavchich M, Russell B, Jaidee A, Chalfein F, Barends M (2008). Amplification of *pvmdr1* associated with multidrug-resistant *Plasmodium vivax*. J Infect Dis.

[25] Ould Ahmedou Salem MS, Ndiaye M, OuldAbdallahi M, M Lekweiry K, Bogreau H, Konaté L (2014). Polymorphism of the merozoite surface protein-1 block 2 region in Plasmodium falciparum isolates from Mauritania. Malar J.

[26] Maffi M. Contribution à la connaissance de la faune anophélienne de la Mauritanie. WHO/Mal/434. World Health Organization, Geneva, 1964. (http://www.who.int/iris/handle/10665/65168).

[27] Hamon J, Maffi M, Grenier P, Ouedraogo CS, Djime D (1966). Notes sur les moustiques de la République Islamique de Mauritanie (Diptera: Culicidae) (2e partie). Ann Soc Entomol Fr.

[28] Dia I, Ba H, Ould Mohamed SA, Diallo D, Lo B, Diallo M (2009). Distribution, host preference and infection rates of malaria vectors in Mauritania. Parasite Vectors.

[29] Coulibaly A (1997). Analyse du système vectoriel du district de Nouakchott.

[30] Ould Ahmedou Salem MS, Mint Lekweiry K, Mint Hasni M, Konate L, Briolant S, Faye O (2013). Characterization of anopheline (Diptera: Culicidae) larval habitats in Nouakchott, Mauritania. J Vector Borne Dis.

[31] Mint Lekweiry K, Basco LK, Ould Ahmedou Salem MS, Hafid JE, Marin-Jauffre A, Bouchiba H (2011). Malaria prevalence, morbidity and drug resistance in Nouakchott, Mauritania. Trop Med Int Health.

[32] WHO (2014). World malaria report 2014.

[33] Anonymous (2013). Health recommendations for travellers (for health professionals). Bull Epidémiol Hebd.

